# Unexpected content of kynurenine in mother’s milk and infant formulas

**DOI:** 10.1038/s41598-022-10075-5

**Published:** 2022-04-19

**Authors:** Marta Marszalek-Grabska, Anna Stachniuk, Paulina Iwaniak, Kinga Gawel, Agata Sumara, Tomasz Kocki, Emilia Fornal, Paweł Milart, Piotr Paluszkiewicz, Waldemar Turski

**Affiliations:** 1 Department of Experimental and Clinical Pharmacology, Medical University of Lublin, Jaczewskiego 8b, 20-090 Lublin, Poland; 2 Department of Bioanalytics, Medical University of Lublin, Jaczewskiego 8b, 20-090 Lublin, Poland; 3 3rd Department of Gynecology, Medical University of Lublin, Jaczewskiego 8, 20-090 Lublin, Poland; 4 Department of General, Oncological and Metabolic Surgery, Institute of Hematology and Transfusion Medicine, Indiry Gandhi 14, 02-776 Warsaw, Poland

**Keywords:** Developmental biology, Molecular biology, Neuroscience, Physiology, Gastroenterology, Medical research

## Abstract

Mother’s milk is widely recommended as complete food for the offspring in earliest postnatal time. However, the knowledge about detailed composition and the physiological role of bioactive components of breast milk is incomplete. Therefore, the aim of our study was to determine the content of kynurenine (KYN) in human breast milk during lactation and to explore the effects exerted by intragastric KYN administration from birth to weaning on physical and psychomotor development of adult rats. We found that KYN is consistently present in human milk and its content gradually increased from day 4 to 28 after delivery and that it is present in commercial baby formulas in amounts noticeably exceeding its physiological range. Animal studies showed that KYN supplementation resulted in a marked elevation of absorptive surface of rat intestine and in enhanced expression of both, aryl hydrocarbon receptor and G protein-coupled receptor 35 in the intestinal tissue in rats. Moreover, we discovered that KYN administration from birth to weaning resulted in neurobehavioral changes in adult rats. Therefore, we postulate that further research is required to thoroughly understand the function of KYN in early developmental stages of mammals and to ensure the safety of its presence in baby food products.

## Introduction

There is a broad consensus that mother’s milk meets all nutritional needs of the newborns promoting their development and maturity. WHO/UNICEF, other relevant scientific bodies and medical practitioners recommend exclusive breastfeeding for about 6 months, and then continuing it while introducing complementary foods until a child is 12 months old or older. When it comes, however, to bioactive components of breast milk, contrary to the common belief, the scientific knowledge is still in its infancy. Only recently, an idea has been put forth to study human milk as a biological system and a call has been made to correctly establish the scientific goal of today’s and future studies. Among the most important directions of the research are: (a) identification of bioactive components of milk at different stages of lactation, (b) explanation of its mechanism of action and developmental importance, (c) ascertaining its physiological ranges in order to evaluate quality of milk and milk substitutes^1^. In response to this challenge and in continuation of our previous study devoted to kynurenic acid (KYNA)^2^, here we propose to focus on the presence of tryptophan (TRP) metabolite kynurenine (KYN) in human breast milk and the effects evoked by supplementation of KYN during maternal feeding in rats.

Kynurenine pathway is the major route for TRP degradation in most mammalian tissues. Through this pathway, 95% of absorbed TRP is transformed to downstream metabolites known as kynurenines^3,4^. KYN is the central metabolite of the kynurenine pathway and its concentration reflects TRP metabolism along the whole kynurenine pathway. It is mainly metabolized to 3-hydroxykynurenine, 3-hydroxyanthranilic acid, quinolinic acid and nicotinamide-adenine dinucleotide (NAD^+^)^5^. KYN is also converted by kynurenine aminotransferases to KYNA. Usually, kynurenines are classified according to their neuroprotective or neurotoxic properties.

Imbalance in the kynurenine pathway is associated with certain neurological and neurodegenerative disorders such as brain ischemia, epilepsy, major depression, Alzheimer’s disease, Huntington’s disease, Parkinson’s disease, multiple sclerosis, HIV associated neurocognitive disorders, or schizophrenia^3,6,7^. Much less is known about the peripheral action of kynurenines. However, in recent years, major progress has been made. It was found that metabolites of TRP, including KYN, KYNA, quinolinic acid, cinnabarinic acid and NAD^+^ are ligands for aryl hydrocarbon receptor (AhR)^8–10^, which is involved in many physiological functions, from early development of embryonic stem cells to adult tissue regeneration, chemical and microbial defense, reproduction and energy metabolism^11^. Moreover, its activation plays an important role in several pathological processes, including inflammation and carcinogenesis^12^.

There have been no reports yet of the content of KYN in particular sections of the gastrointestinal tract, as proven for KYNA as a ligand for orphan G protein-coupled receptor (GPR35)^13^, which reaches the highest concentration in the distal part of the gastrointestinal tract enabling their interaction^14^. A high peripheral concentration of KYN has, however, been found in the rat liver, where KYN is converted from TRP by the tryptophan 2,3-dioxygenase (TDO), which catabolizes the majority of dietary TRP for the maintenance of its basal serum level^15^.

Imbalance in the kynurenine pathway in response to inflammatory conditions leads to an upregulation of indoleamine 2,3-dioxygenase (IDO). In this aspect, KYN is more likely to be metabolized to 3-hydroxykynurenine and quinolinic acid^16,17^. Fate and action of KYN with a special emphasis on periphery has recently been reviewed by Marszalek-Grabska et al.^18^.

The presence of KYN in human milk was previously mentioned by O’Rourke et al.^19^ and Gómez-Gallego et al.^20^ but was not studied in detail. Therefore, the aim of our study was to determine the content of KYN in human breast milk during 4 weeks of lactation as well as in commercial baby formulas used in the earliest postnatal time of feeding. Additionally, we tested the effects exerted by KYN administered during breastfeeding on the postnatal gastrointestinal tract maturation. Due to known blood–brain barrier permeability of KYN a psychomotor activity was estimated in adult rats exposed to this drug administered intragastrically in the suckling time.

## Results

### Investigation of the presence and content of tryptophan and kynurenine in human milk

**Figure 1:**
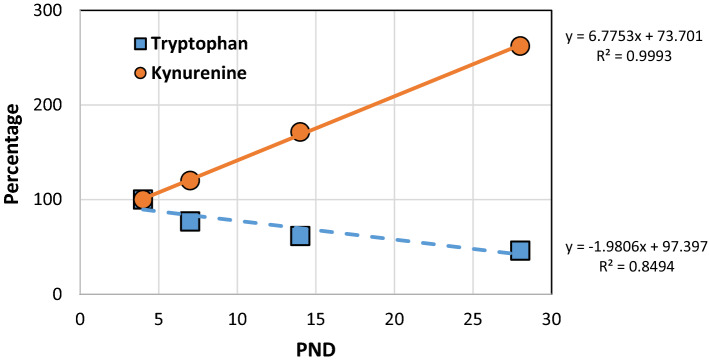
Graphical presentation of tryptophan and kynurenine content in human milk—trend analysis. Data representing content of compounds are percentage of values detected at PND 4. A straight line depicts a trend in the data described by a first order equation (y = ax + b). R^2^ is a correlation coefficient value. *PND* postnatal day.

On day 4 the content of TRP and KYN in breast milk was 1.343 and 0.031 μg/mL, respectively (Supplementary Table S1). TRP concentration was decreased linearly by 50% from day 4 to day 28 (Supplementary Table S1, Fig. 1). On the contrary, KYN concentration increased rapidly up to 250% on day 28 (Supplementary Table S1, Fig. 1).

### Investigation of the presence and content of tryptophan and kynurenine in baby formulas

Content of TRP and KYN was measured in eleven, 9 powdered and 2 liquid, baby formulas available commercially. Content of TRP and KYN in powdered formulas ranged from 2.2 to 9667.6 µg/g and from 0.2 to 173.4 µg/g, respectively (Table 1). Concentration of TRP and KYN in baby food prepared according to recommendations of producers ranged from 0.2 to 1482.4 µg/mL and from 20.0 to 28,289.3 µg/mL, respectively (Table 1).

**Table 1:** Content of tryptophan and kynurenine in baby formulas.

Commercial product	Suggested for ages	Form	Tryptophan		Kynurenine	
			µg/g of powder	µg/mL of ready to use liquid	µg/g of powder	ng/mL of ready to use liquid
Aptamil Pepti 1	From birth	Powder	1505.4	225.8	1.7	255.0
Aptamil Pepti 2	From 6 months	Powder	1552.0	248.3	1.7	272.0
Enfamil Premium A.R. 2	From 5 months	Powder	2.2	0.3	0.2	28.7
Hipp Combiotik 1	From birth up to 6 months	Powder	155.3	19.1	0.3	36.9
Nutricia Bebilon HA Pro Expert 2	From 7 months	Powder	45.0	7.4	14.5	2368.3
Nutricia neocate 0–12 months	From birth up to 12 months	Powder	9667.6	1482.4	0.3	46.0
Nutramigen Puramino for infants from birth	From birth	Powder	8895.4	1334.3	0.9	135.0
Nutramigen LGG Lipil 1	From birth	Powder	3610.1	541.5	173.4	26,010.0
Nutramigen LGG Lipil 2	From 6 months	Powder	4245.9	693.5	173.2	28,289.3
Bebilon Nenatal premium pronutra	From birth	Liquid	–	0.5	–	20.0
Nestle NAN Optipro plus 1	From birth	Liquid	–	0.2	–	20.0

Liquid ready to use was prepared according to recommendation of producer. Data are presented as a mean value of 3 repetitions. Standard deviation was below 5% for kynurenine and below 6.5% for tryptophan.

### Investigation of the effect of kynurenine administration on its content in blood and liver of 22-day old rats—does it accumulate?

Intragastric administration of KYN from day 2 until day 21 did not result in a significant alteration of KYN content in blood plasma and liver tissue of male and female 22-day old rats (Supplementary Table S2) pointing against accumulation of the drug in the rat body.

### Investigation of the effect of kynurenine administration on morphology of jejunum of 22-day old rats

Histomorphological analysis of jejunum obtained from 22-day old rats of both sexes treated with KYN during maternal feeding revealed increased thickness of mucosa and enhanced height of villi without any change in their width. Calculated absorptive surface of intestine was markedly elevated (Table 2).

**Table 2:** Effect of kynurenine administration during maternal feeding on histomorphometric parameters of jejunum of 22-day old rats.

Gender	Group	Intestine absorptive surface (µm)	Mucosa thickness (µm)	Villus height (µm)	Villus width (µm)
Male	Control	5.8 ± 2.0	254.6 ± 45.1	150.0 ± 49.2	56.6 ± 17.8
	KYN	9.8 ± 2.9*	326.3 ± 78.1*	246.5 ± 71.7*	58.3 ± 15.3
Female	Control	6.3 ± 2.3	244.0 ± 61.0	150.5 ± 67.0	45.5 ± 20.1
	KYN	11.7 ± 2.4*	342.9 ± 72.0*	279.4 ± 49.4*	57.6 ± 11.7

Data are presented as a mean ± SEM, number of subjects in each group = 10, *P < 0.05 vs respective Control, *t*-Student test. *KYN* kynurenine.

### Investigation of the effect of kynurenine administration on expression of receptors affected by kynurenine and its metabolite, kynurenic acid

**Figure 2:**
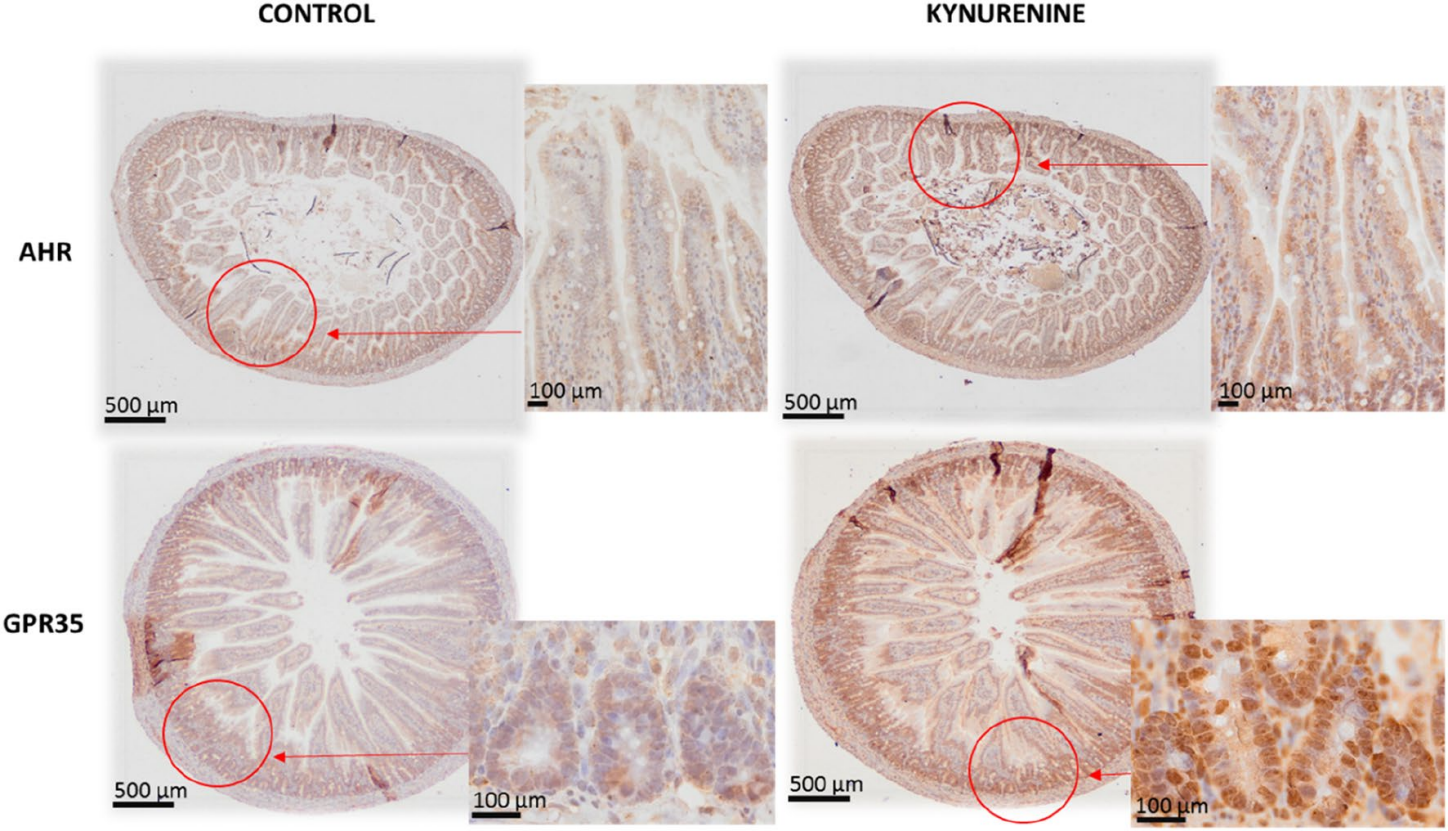
Representative photomicrographs of the immunohistochemical reactions for AhR and GPR35 in the jejunum of rats treated with kynurenine during maternal feeding. Sections were developed in 3,3′-diaminobenzidine tetrahydrochloride (DAB); counterstaining was performed with Mayer’s hematoxylin.

Immunohistochemical analysis revealed that AhR (KYN and KYNA molecular target) and GPR35 (KYNA molecular target) are highly expressed in the mucosa of the jejunum of rats at postnatal day (PND) 22 (Fig. 2). The expression of both, GPR35 and AhR protein was higher in the crypts and epithelial villi of KYN-treated rats compared to control saline-treated rats. However, when the localization of GPR35 staining was analyzed separately for villi and crypts, a stronger dye accumulation was noted in the bottom of the crypts, where stem cells are dominant. In contrast to GPR35, the expression of AhR receptor was distributed uniformly in the cell cytoplasm along the entire length of the epithelial villi (Fig. 2).

### Investigation of the effect of kynurenine on body mass gain of young and adult rats

Intragastric administration of KYN from PND 2 until PND 21 affected body mass gain measured up to PND 63 of life neither in male nor in female rats (Supplementary Fig. S1).

### Investigation of the effect of kynurenine administration during maternal feeding on locomotor activity of adult rats

To assess the effect of intragastric administration of KYN from PND 2 until PND 21 on the behavior of adult 56-day old rats, 5 parameters of motor activity covering horizontal and vertical movements, and stereotypy were analyzed. It was found that KYN administration during maternal feeding did not affect spontaneous locomotor activity of adult male and female rats (Supplementary Figs. S2, S3). To further investigate the susceptibility of the adult rats to stimulants dizocilpine and amphetamine were administered immediately before the locomotor activity test. It was found that KYN administration during maternal feeding reduced susceptibility of female but not male rats to the action of dizocilpine (Supplementary Figs. S2, S3). It was established that KYN administration during maternal feeding enhanced susceptibility of male but not female rats to the action of amphetamine (Supplementary Figs. S2, S3).

### Investigation of the effect of kynurenine administration during maternal feeding on anxiety-like and depressive-like behavior of adult rats

KYN administration during maternal feeding did not change the number of entries into the open arms, the time spent by the rats in the open arms and the number of total entries into both open and closed arms in adult rats of both sexes (Supplementary Fig. S4). Thus, the treatment with KYN during maternal feeding had no effect on anxiety-like behavior as compared to the respective control groups.

KYN administration during maternal feeding did not change the immobility time in the Porsolt swim test in adult rats of both sexes. Thus, the treatment with KYN during maternal feeding had no effect on depressive-like behavior as compared to the respective control groups (Supplementary Fig. S5).

### Investigation of the effect of kynurenine administration during maternal feeding on recognition memory, spatial memory and associative learning of adult rats

KYN administration during maternal feeding had no effect on novel object recognition memory measured in adult rats of both sexes. During the training session, the subject rats spent a similar period of time exploring two identical objects. During the testing session performed 24 h later no significant difference in the exploration of the novel and familiar object measured as discrimination index (Supplementary Fig. S6A,B) and total exploration time (Supplementary Fig. S6C,D) was determined.

**Figure 3:**
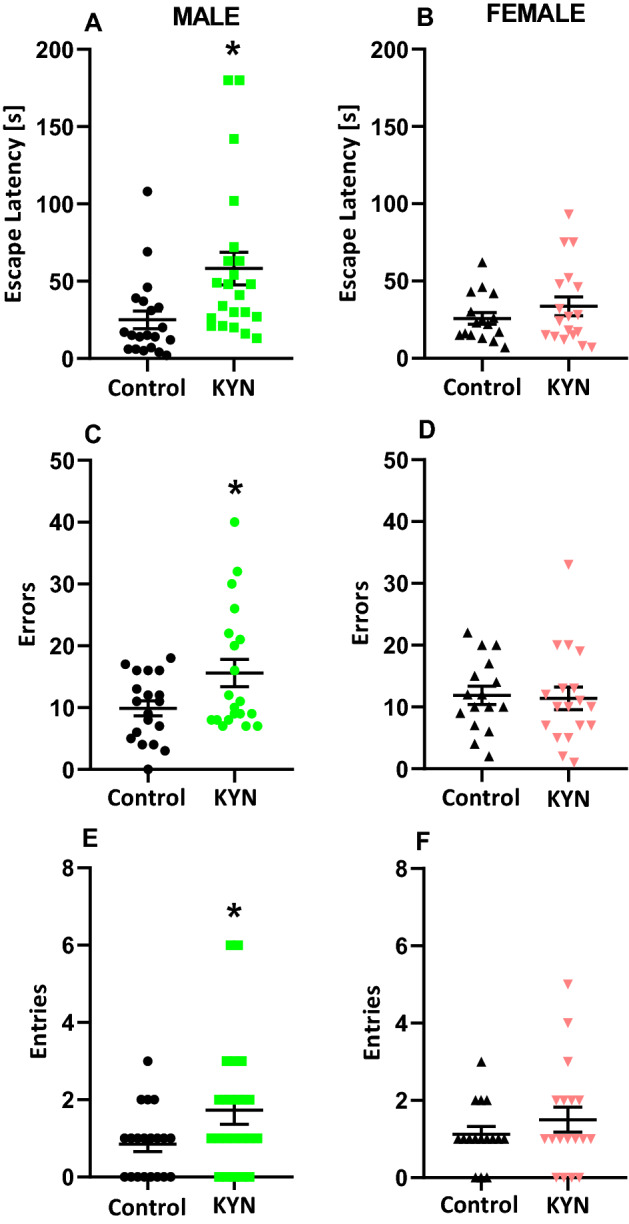
Effect of kynurenine administration during maternal feeding on reversal learning of adult rats. Escape latency (**A**,**B**), errors committed (**C**,**D**), entries into the previous escape location (**E**,**F**). Data are presented as a mean ± SEM, number of subject = 9–11 per group, *P < 0.05 vs respective Control, Student *t*-test. *KYN* kynurenine.

Reversal learning trail in the Barnes maze was conducted 24 h after completing the training which lasted 3 consecutive acquisition days. Male rats exposed to KYN during maternal feeding had significantly higher escape latencies (Fig. 3A), committed more errors (Fig. 3C) and attempted more entries into the previous escape location (Fig. 3E). Female rats exposed to KYN during maternal feeding did not differ from their respective control counterparts (Fig. 3).

**Figure 4:**
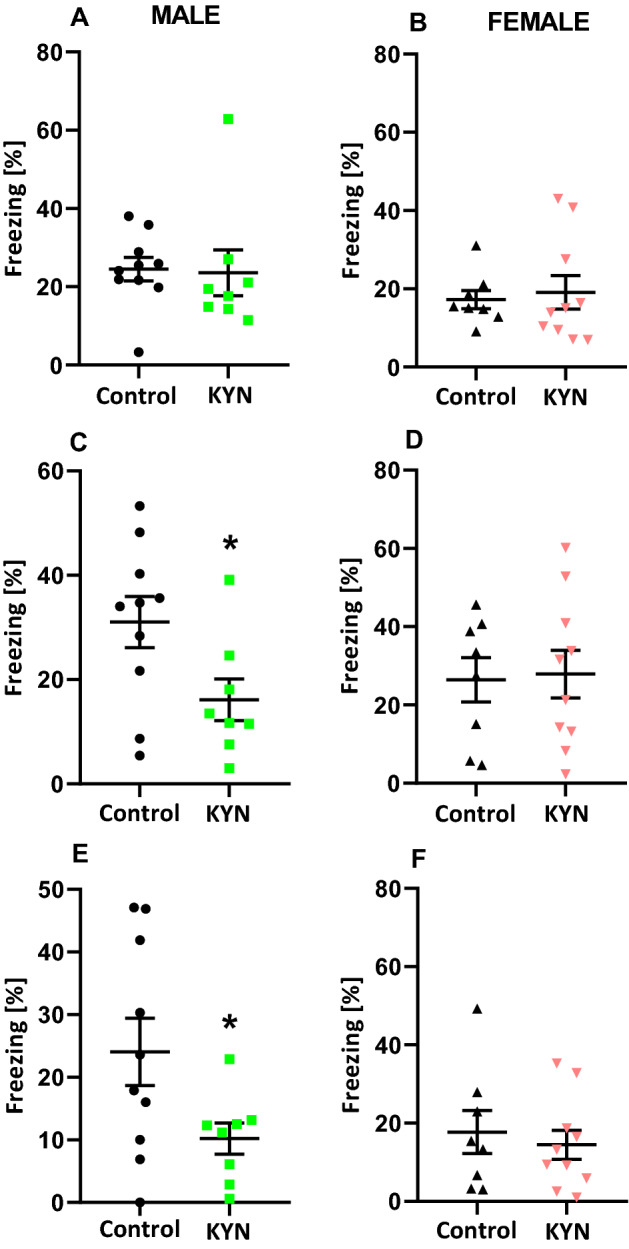
Effect of kynurenine administration during maternal feeding on associative learning of adult rats. Training of the fear conditioning test (**A**,**B**), contextual fear conditioning test (**C**,**D**), cue fear conditioning test (**E**,**F**). Results are expressed as mean ± SEM. ^*^P < 0.05 vs respective Control. N = 8–10 per group. *KYN* kynurenine.

There were no significant differences between the groups during the training in the fear conditioning test (Fig. 4A,B). However, the contextual fear conditioning test indicated that male rats exposed to KYN during maternal feeding exhibited decreased freezing response (Fig. 4C). A similar effect was noted 24 h later, as the cue fear conditioning test indicated that male rats exposed to KYN during maternal feeding had a reduced freezing response (Fig. 4E). There was no difference between female rats treated with KYN during maternal feeding and their female control counterparts in the fear conditioning test (Fig. 4D,F).

## Discussion

The growing data collected from chemical, immunological and biological analyses of mammals’ milk foster artificial formula construction enabling safe nutritional support of the offspring. Amino acid concentration changes in human milk during breastfeeding were also widely analyzed and finally summarized by Zhang et al.^21^. In the first two months of lactation the total content of amino acids declined. Detailed analyses showed the decrease in 14 amino acids including TRP and unchanged concentration of 3 others. Only two amino acid concentrations in human milk increased during breastfeeding but this phenomenon has not been elucidated further^21^.

Formerly, we identified KYNA, as a constituent of breast milk. KYNA concentration in human milk increases more than 13-times during the first six months of breastfeeding. Finally, its biological activity was confirmed in developing animals^2^. Here, we found that a KYNA precursor—KYN is consistently present in milk collected from breastfeeding women and gradually increased from day 4 to 28 after delivery. Interestingly, at first month of lactation TRP concentration in breast milk dropped nearly half as much. The increase in KYN concentration in human milk between first and second week of lactation was signalized previously, however without changes of TRP content^19^.

The stable increase in KYN concentration in milk during breastfeeding was independent of non-restrictive diets used by the studied mothers. Therefore, we assume that mothers’ milk KYN content is regulated and plays an important role in offspring postnatal maturation. The primary exposition of neonatal digestive tract on milk KYN suggests developmental effect on intestinal postnatal integrity during breastfeeding.

KYN is a ligand of AhR that is abundantly distributed in intestinal mucosa, where it plays an important role in maintaining the epithelial barrier. Moreover, its involvement in inflammatory and autoimmunity processes as well as in gut microbiota composition were widely recognized^22–24^.

It was found that the Ahr (−/−) knockout mice^25^ as well as GPR35 (−/−) knockout mice^26^ aggravate intestinal inflammation in colitis model. The ability of intestinal epithelial cells to remodel and to heal intestinal wounds depends on AhR activity and ligand bioavailability by the regulation of interleukine 10 receptor expression on intestinal epithelia^27^. It is very probable that KYN interaction with intestinal lymphoid tissue attenuates inflammatory response on intestinal microbiota and elevates environmental antigen toleration. Moreover, TRP administration markedly mitigates intestinal inflammatory response in mice and improves intestinal immune defense^28^. According to KYNA concentration in human milk presented previously^2^ and the current findings of KYN content we suggest a hypothesis that kynurenine pathway metabolites play an important role in postnatal intestinal maturation and environmental antigen tolerability during breastfeeding.

The role of AhR in the development of intestine has been insufficiently studied to date. In neonatal AhR (−/−) knockout mice the loss of intestinal homeostasis and increased bacterial translocation were found^29^. Here, we found that in 22-day old rats AhR staining is present and uniformly distributed in the cytoplasm of the cells of epithelial villi. Increasing AhR density in KYN exposed suckling rats can be interpreted as a result of ligand-receptor interaction. Thus, we found that KYN supplementation resulted in increased thickness of mucosa and enhanced height of villi without any change in their width. Consequently, an absorptive surface of intestine was markedly elevated in KYN-treated rats. Moreover, the expression of both, AhR and GPR35 protein was higher in the crypts and epithelial villi of KYN-treated compared to control saline-treated rats. These morphological changes, however, were not accompanied with altered body mass gain in both young and adult rats. KYN and downstream metabolites are implicated in the regulation of a number of host systemic biological processes involving neurotransmission, inflammation, and the immune response. However, previous reports revealed that some of them appear to exert specific effects in the gut. For instance, KYNA concentration increases gradually along the gastrointestinal tract^14^ and KYNA exhibits mucosal protective and immunoregulatory effects, probably through GPR35 mostly expressed in epithelial and immune cells^13^. Both, KYN and KYNA are ligands for AhR. It has been indicated that the diet rich in AhR ligands links to the expression of genes responsible for enterocyte differentiation, the turnover rate and epithelial cells lineage fate^30^. Further, epithelial IDO1, an enzyme catalyzing metabolism of TRP to KYN, was found to non-enzymatically modulate AhR to inhibit activation of Notch1 resulting in increased differentiation of secretory cells and thickening of intestinal mucus layer^31^. This finding is in agreement with our result, which confirms the importance of AhR in controlling intestinal structure and function.

AhR is an environmentally-sensitive and evolutionary old transcription factor belonging to the Per-ARNT-Sim-basic helix-loop-helix protein family with partially identified targeted genes. The significant overexpression of AhR after KYN treatment was previously observed in endometrial stromal cells under in vitro decidualization. Moreover, AhR target genes like *CYP1A1* and *CYP1B1* also overexpressed and suggested KYN-induced activation of AhR-related signal transduction^32^.

Activation of AhR by agonist’s interaction in ligand biding pocket is connected with translocation of active complex into nucleus and regulates transcription. The transcriptional changes by the activating AhR are ligand and cell specific. According to the results obtained from colon cancer cell lines, KYN was identified as endogenous ligand of AhR^9^. The data indicate that activation of AhR by KYN elicits a gene expression program regulating cell proliferation and migration. The elevation of AhR density can be connected with proliferative activity^33^ after complex formation with KYN. Our histomorphological analyses of rat intestine presented a significant elevation of villi length in small intestine after KYN ingestion. The increase in villi length was independent of villi width and thus, finally, the total surface area of mucous significantly increased in KYN treatment group. The normal intestinal epithelial cells on lengthened villi suggested hyper-proliferative effect of KYN during postnatal intestinal development. Similar effect with significant villi elongation has been reported recently in mice after TRP administration^28^. The proliferative activity is probably independent of *Wnt-β-catenin* signaling. The deletion of AhR in intestinal epithelial cells resulted in unrestricted intestinal stem cell proliferation and impaired differentiation of intestinal epithelial cells across the villi^34^. AhR activated complexes protecting intestinal stem cells niche and restored barrier homeostasis without a significant sign of dysplastic lesions. The normal villi width suggests no effect of KYN on stromal cells; however, pro-angiogenic action during elongation of villi should be considered. The angiogenic activity connected with the elevation of KYN/TRP ratio in serum and tumor microenvironment as well as the overexpression of IDO1/TDO, which regulate kynurenine pathway were observed in advanced malignancies^35^.

AhR activation may also be involved in the occurrence of obesity; however, this mechanism is not fully understood. One hypothesis assumed that it might be related to the deregulation of lipid, glucose and NAD^+^ homeostasis through NAD^+^—and sirtuin^36^. Recently, Rojas et al*.*^37^ have indicated that KYN caused body mass gain in mice with a low-fat diet, developed a fatty liver and hyperglycaemia and increased liver *Cyp1b1* and *Scd1* gene expression. The authors concluded that AhR activation by KYN may be necessary but insufficient for the development of obesity and that excess caloric consumption is also required for a full manifestation of obesity^37^. The above results are in agreement with our results in which we did not observe any weight gain in the animals administered KYN and fed a normal diet. A cautious hypothesis can be proposed that the role of KYN is to compensate for nutritional deficiencies, e.g. morphological changes in the intestine, but this requires further research.

The behavioral experiments performed on adult rats revealed significant differences between the rats treated with KYN during suckling period and the saline-treated counterparts. We discovered that KYN administration in suckling period of life reduced susceptibility of female but not male rats to the action of dizocilpine and enhanced susceptibility of male but not female rats to the action of amphetamine. Both drugs are used to mimic psychotic-like conditions in experimental pharmacology. Furthermore, cognitive flexibility determined in the Barnes maze task and the contextual fear conditioning were significantly impaired in males but not females. Moreover, there was no effect of KYN administered in suckling period on spontaneous locomotor activity, recognition memory, anxiety-like and depressive-like behavior in adult rats. The reason for late manifestation of KYN-administration in suckling period is unknown. We found that KYN does not accumulate in blood plasma and liver tissue of the rats as examined 24 h after the last dose; thus, ordinary intoxication mechanism can be ruled out. Moreover, clearly gender-dependent differences registered in behavioral tasks point against overall KYN intoxication.

Our study is perfectly in line with research conducted by Liu et al.^38^ despite substantial methodological differences. KYN was administered in mice intraperitoneally (i.p.) twice daily from PND 7 to PND 17. KYN-treated neonatal mice displayed unaffected basal locomotor activity and enhanced locomotor responsiveness to amphetamine, impaired working memory in the trace fear conditioning task and no sign of anxiety at age 5–6 months^38^.

In the rats treated i.p. with KYN at PND 7–10 and tested at age 70 days, significantly fewer social interactions were found. However, no significant alteration in fear conditioning and attentional orienting tests were observed^39^. These data demonstrate that behavioral effects evoked by intragastric KYN administration can be replicated by intraperitoneal application of the drug. Thus, it can be concluded that central nervous system derangement depends on KYN absorbed from digestive system rather than on its action in the intestine. Indeed, KYN is readily transported through the intestinal barrier and penetrates blood–brain barrier unlike most of its metabolites. It is not known whether KYN exerts its action directly or by metabolites. Some authors postulate that KYN-mediated effects are in fact produced by the elevation of KYNA^40^. However, the involvement of other neuroactive KYN metabolites in the effects evoked by KYN administration cannot be excluded either^18^.

Interestingly, our behavioral findings are consistent with the data presented in the series of publications in which rats were exposed to parenterally infused KYN pre- and postnatally [see for review^40^]. Therefore, for the first time we provide evidence that alimentary administration of KYN from birth to weaning may affect brain functioning in adults.

Since we found a stable concentration of KYN in human breast milk we were deeply concerned about the presence and the content of this compound in baby food formulas. The vanishingly low concentration of KYNA in commercial baby food formulas was described previously^2^. Unexpectedly, here we detected extremely different quantities of KYN, the precursor of KYNA. The content of KYN ranged from 0.02 to 28 µg/mL in the prepared liquid mixtures. The highest amount of KYN was found in extensively hydrolyzed baby formulas (Nutramigen). It should be emphasized that in most non-hydrolyzed products its content was close to that in human milk. Concurrent determination of TRP in infant formulas was undertaken. It was found that its amount in baby food products did not parallel that of KYN. The highest concentration of TRP was found in Nutricia neocate and Nutramigen Puramino amounting to 1.5 and 1.3 mg/mL, respectively. The relative high concentrations of TRP ranging from 0.07 mg/mL^41^ to 4.5 mg/mL^42^ were reported in extensively hydrolyzed infant formulas.

Although extensive hydrolyzed baby formulas have been used for a long time now the reports on their cognitive developmental effects are rare. According to Mennella et al.^43^ values representing gross motor skills and visual reception were significantly higher but that receptive language defining and the ability to process linguistic input were lower in children fed extensive protein hydrolyzate formulas in comparison to children fed cow milk formula^43^. The significant differences in body weight and composition and the difference in only one out of 8 parameters of intellectual skills between children fed breast milk, cow’s milk-based or soy protein-based formulas were reported at the age of 6 years^44^. The effect of infant feeding mode on childhood cognition and language discloses that significant differences were noted in children not younger than 36 months. A significant effect of sex was also found; however, the final conclusion was that sexual dimorphism in child cognitive and language development and skill acquisition is not a substantial concern^45^. In our opinion, despite no alarming indications in the literature, this issue deserves particular attention. In future analyses a special focus should be given to the age of participants, division of studied groups by gender and to the selection of proper, sensitive tests.

Taken all together, we found that KYN is present in human milk and commercial baby formulas and we indicated the presence of its target receptors AhR in digestive tract of young animals fed exclusively on their mother’s milk. The interaction of dietary KYN and intestinal epithelial cells AhR affect postnatal gastrointestinal mucosa remodeling. Moreover, we show that oversupply of KYN in the diet during early neonatal life can affect functional brain development and contribute to adverse functional changes in adulthood at least in rats. Based on these results we postulate comprehensive control of the micro-content of KYN in baby food products in order to exclude potential detrimental neurobehavioral effects. Further research is required to thoroughly understand the function of KYN in the early process of development.

## Methods

### Human study

Material for the study was breast milk from 36 healthy parturients collected within 4 weeks after the delivery. Informed consent was obtained from all parturients. The protocol applied had the approval of the Bioethics Committee of the Medical University of Lublin, Poland (KE-0254/168/2009). The mothers received instructions on the method of breast milk collection.

### Sample collection

Breast milk was harvested in four batches: on 4th, 7th, 14th and 28th day after delivery. After the first breastfeeding of the day, samples in the amount of 5 mL were collected with the use of breast pumps into plastic containers and stored in a fridge for not more than 3 h. Subsequently, they were transported to the lab in human tissue transport boxes, aliquoted and stored at − 80 °C. All the procedures were performed in accordance with the applicable regulations and guidelines.

### Animal study

Wistar rats of both sexes were used in the experiments. The animals were maintained under standard laboratory conditions (12 h light/dark cycle, room temperature 21 ± 1 °C) with free access to tap water and laboratory chow (Sniff Spezialdiäten GmbH, Soest, Germany). Each experimental group consisted of 7–12 animals. All the experiments were conducted according to the National Institute of Health Guidelines for the Care and Use of Laboratory Animals and to the European Community Council Directive for the Care and Use of Laboratory Animals of 22 September 2010 (2010/63/EU). The study was approved by the Local Ethics Committee for Animal Experiments, Lublin, Poland (No 49/2019 and 38/2020). The study was conducted in accordance with ARRIVE guidelines.

### Study design

**Figure 5:**
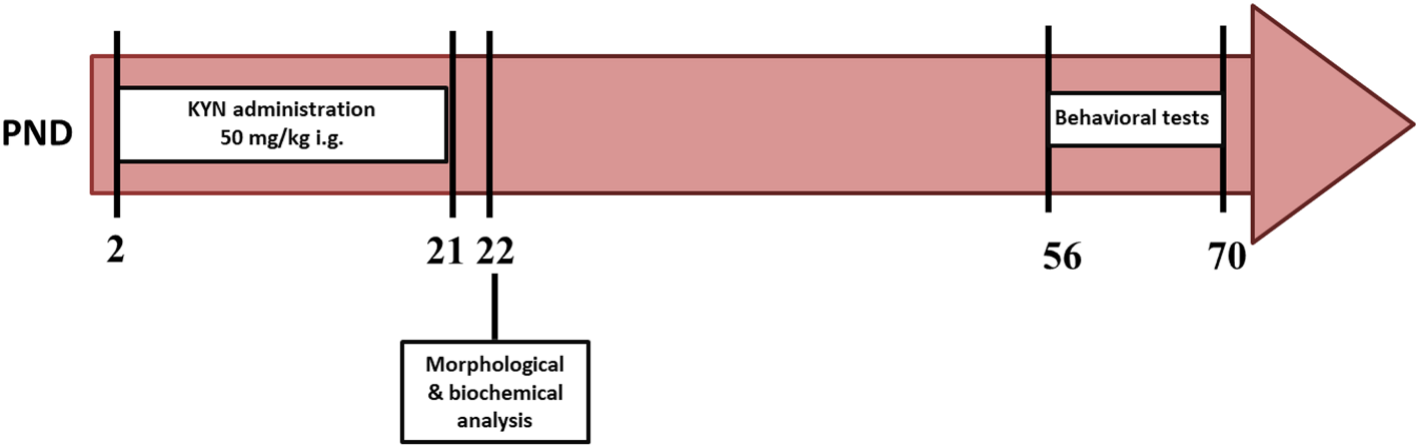
Schematic representation of experimental study design and analysis. *PND* postnatal day, *i.g.* intragastrically, *KYN* kynurenine, *PND* postnatal day.

The study design is shown in Fig. 5. KYN was administered intragastrically via polyurethane cannula (FTP, Instech, 0.5 × 0.9 mm) at the dose of 50 mg/kg in a volume of 5 mL/kg to male and female offspring once a day from PND 2 to PND 21. Control groups received saline (0.9% NaCl) at the same time points. The animals stayed undisturbed with their dams from birth to PND 21, except for short intervals for substance administration. They were weighed every day before injection and additionally on PND 28, 35, 42, 49, 56, 63. Male and female offsprings were used in biochemical and behavioral experiments.

### Tissue sampling and preparation

Plasma and liver were collected at PND 22. The animals were decapitated, the plasma was obtained by a centrifugation of the blood at 1620*g* for 30 min (temp. 4 °C) and the liver was rapidly removed and frozen on dry ice. The samples were stored at − 80 °C until further processing. The blood plasma was deproteinated with 8% perchloric acid. The samples were vortexed, kept at 4 °C for 10 min, and centrifuged at 20,598×*g* for 30 min at 4 °C. The liver samples were dissected on wet ice, weighed and sonicated in ultrapure water (1:3 w/v). Then the samples were deproteinated with 8% perchloric acid, centrifuged at 20,598*g* for 30 min at 4 °C and prepared for analysis using high performance liquid chromatography (HPLC).

### Infant nutritional formulas

Eleven feeding formulas designed for alternative feeding of human infants were commercially purchased (for brand names see Table 1). The samples of powdered milk formulas were prepared according to the producers’ recipes.

### Chemicals

l-Kynurenine sulfate, d-amphetamine sulfate and dizocilpine (5-methyl-10,11-dihydro-5H-dibenzo [a,d] cyclohepten-5,10-imine) were obtained from Sigma-Aldrich, USA. All drugs were dissolved in saline and prepared *ex tempore*. pH of KYN solution was adjusted to 7.0 with 0.1 N NaOH. D-amphetamine sulphate (1.0 mg/kg) and dizocilpine (doses 0.075, 0.15 and 0.3 mg/kg) were administered i.p. in a volume of 2 mL/kg. In all the cases, the control animals received corresponding saline injection. HPLC reagents were purchased from J.T. Baker Chemicals or Sigma-Aldrich. The analytical stable isotope-labeled standards of [^2^H_5_]-tryptophan and [^13^C_6_]-kynurenine were purchased from Alsachim (Illkirch-Graffenstaden, France). Acetonitrile and methanol for LC/MS analyses, of Optima® LC/MS grade, were purchased from Fisher Chemical (Waltham, MA, USA). Formic acid (LC/MS grade) and diethyl ether (HPLC grade) were purchased from Merck KGaA (Darmstadt, Germany). Titan Syringe Filters RC, 0.2 μm were supplied by Thermo Scientific (Waltham, MA, USA). The ultra-pure water was obtained from Millipore Direct-Q3-UV purification system (Merck KGaA, Germany).

### Morphometric analysis

Samples of small intestine from each PND 22 rat were collected and fixed in 4% buffered formaldehyde (pH 7.0) for 12 h, dehydrated in graded ethanol solutions, cleared in xylene and embedded in paraffin^46^. Cross sections of 4 μm thick were cut with a microtome (Microm HM 360, Microm, Walldorf, Germany) from every sample of the small intestine (jejunum) material. For visualization of selected structures Goldner’s trichrome method was used as described by Dobrowolski et al.^47^. Microscopic images of each slice were taken using a fully-motorized and automated inverted Optic Microscope IX83 confocal microscope (Olympus, United States) equipped with a color digital camera. The analysis of collected images was performed using graphic analysis software ImageJ 1.52 (National Institute of Health, USA). The following parameters for the small intestine histomorphometry were analyzed: mucosa, submucosa, and myenteron thickness, crypt depth and width, the number of crypts, villus height and width, the number of villi *per* millimeter of mucosa, and the number of enterocytes and goblet cells *per* 100 μm of villus epithelium, villus epithelium thickness and small intestinal absorptive surface as well as the villi length to crypt depth ratio were calculated.

### Determination of tryptophan and kynurenine levels in milk and infant nutritional formulas

Samples of powdered infant formula (5 g) were suspended in 30 mL of milliQ boiled water cooled to 40–50 °C according to the manufacturer’s recommendation. Next, the samples were shaken by hand for 2 min. 2.5 mL of suspended infant formula, liquid infant formula and mothers’ milk were aliquoted to 15 mL centrifuge tubes, isotopically labelled internal standards—[^13^C_6_]-kynurenine and [^2^H_5_]-tryptophan—were added, and the samples were vortexed with 1.25 mL of diethyl ether for 5 min. Next, the samples were centrifuged at 12,100×*g* for 5 min. The top layer was discarded. The bottom layer was collected to a 2 mL syringe and transferred to a new centrifuge tube. 4 mL of methanol was added, the samples were shaken and placed at − 4 °C for 30 min, then centrifuged at 12,100×*g* for 5 min. The supernatants were filtered through a 0.2 μm syringe filters into chromatographic vials and subjected to LC/MS analyses. The determination of TRP and KYN was performed by an Agilent Technology 1290 Infinity series HPLC coupled to an accurate mass quadrupole time-of-flight 6550 iFunnel Q-TOF mass spectrometer equipped with a Jet Stream ion source. The detailed method parameters are reported in our previous paper^48^. Briefly, Zorbax Extend-C18 RR HT (2.1 × 100 mm 1.8 μm) column and a gradient of 0.1% formic acid in water and 0.1% formic acid in acetonitrile at the flow rate of 0.4 mL/min were employed. The quantification was performed on protonated ions [M + H]^+^. For KYN and TRP ions of *m/z* 209.0921 and 205.0972 were extracted, respectively. For isotopically labeled internal standards, ions of *m/z* 215.1122 and 210.1285 were extracted for [^13^C_6_]-kynurenine and [^2^H_5_]-tryptophan, respectively. The method precision expressed as relative standard deviation was below 5% for KYN and below 6.5% for TRP.

### Determination of kynurenine in animal tissue

KYN concentrations were measured according to method of Zhao et al*.*^49^. In brief, the studied substances were analyzed by HPLC system (The UltiMate 3000 Analytical systems (Thermo Fisher Scientific, Waltham, MA, United States)). The samples were separated on analytical column (Agilent HC-C18; 250 × 4.6 mm, inner diameter). The mobile phase was composed of 20 mmol/L NaAc, 3 mmol/L ZnAc2, and 7% acetonitrile. It was pumped at a flow-rate of 1 mL/min and the volume per injection was 100 μL. The wavelength of UV detector was set at 365 nm for KYN determination. Chromeleon software was used to control HPLC system and record chromatographic data.

### Behavioral tests

Behavioral tests were performed in three separate cycles on three different groups of rats aged 56, 63, and 70 days, respectively. There was a 7-day interval between the individual tests. Behavioral tests were carried out according to the principle: from the least to the most invasive, in order to minimize the risk of a possible negative impact of the test conditions/procedure on the results obtained in the next test (Fig. 5). All behavioral tests were performed by highly trained experimenters, blind to treatment groups.

During the first cycle of behavioral experiments, Wistar rats were subjected to a spontaneous locomotor activity test on day 56, an elevated plus maze test on day 63 and a forced swim test on day 70 of age of animals.

During the second cycle of behavioral experiments, Wistar rats were subjected to a dizocilpine-induced locomotor activity test on day 56, a novel object recognition test on day 63 and a contextual and cued fear conditioning test on day 70 of age of animals.

During the third cycle of behavioral experiments, Wistar rats were subjected to a dizocilpine-induced locomotor activity test on day 56, a Barnes maze test on day 63, and an amphetamine-induced locomotor activity test on day 70 of age of animals.

### Locomotor activity

The locomotor activity of each individual rat was recorded using a photocell apparatus (Digiscan, Omnitech Electronics, USA). The animals were individually placed in a plexiglass box (dimensions: 30 cm × 30 cm × 40 cm), in a sound-attenuated experimental room, under moderate illumination (5 lx). Exploratory activity (0–15 min) and spontaneous activity (15–30 min) of the rats individually placed in a new area was measured by vertical and horizontal infrared sensors. Moreover, the following parameters were measured: total distance, number of movements, vertical activity and the number of stereotypy episodes.

### Amphetamine- and dizocilpine-induced locomotor activity

The animals received a challenge dose of amphetamine (1.0 mg/kg, i.p.) or dizocilpine (0.075, 0.15 and 0.3 mg/kg, i.p.). Immediately after the injection, the rats were placed in the photocell apparatus (Digiscan, Omnitech Electronics, USA) and the distance traveled as well as other parameters were recorded for 60 min as described above.

### Elevated plus-maze

The plus-shaped maze was made of wood and positioned on a height of 50 cm above the floor, in quiet laboratory surroundings. Two opposite arms were open (50 × 10 cm) and the other two were enclosed with walls (50 × 10 × 40 cm). The experiment was carried out in a quiet, darkened room with a constant light of 100 lx, located 80 cm above the maze, and directed towards the apparatus. The experiment was initiated by placing the rat in the center of the plus-maze, facing the open arm, after which the number of entries, the time spent in each of the two arms, the distance traveled in the open arms, as well as the total distance traveled were recorded for a period of 5 min. Each “arm entry” was recorded when the rat entered the arm with all four paws. The maze was carefully cleaned with 10% ethanol solution after each test session. The experiment was videotaped and a highly trained experimenter made all the recordings manually.

The anxiolytic/anxiogenic effect for each rat was measured as: (a) time spent in the open arms as a percent of total time spent exploring both open and enclosed arms, (b) the number of entries into the open arms as a percent of the total number of entries into both the open and the enclosed arms. Locomotor activity of each rat was evaluated as the total number of entries into both the open and the enclosed arms of the apparatus.

### Forced swim test

The rats were placed in a glass cylinder (height 50 cm, diameter 20 cm) containing 30 cm of water, that was maintained at 25 °C. Two swim sessions were conducted: an initial 15 min pretest followed 24 h later by a 5 min test. The total duration of immobility (that is, the time during which the rat made only minimal movements in order to stay afloat without any attempt to escape, or it showed only slow movements so as to keep its head above the water) was recorded during the test.

### Novel object recognition

The novel object recognition test was carried out in the same plexiglass square box (63 cm long × 44.5 cm high × 44 cm wide) in a dimly lit (30 ± 5 lx) testing room. One day before a training session the rats were habituated for 15 min to the testing arena. Then, all the rats were given two 3-min sessions (a training session and a testing session). During the training session (the first session, T1A, familiarization phase), the rats explored two identical objects (A1 and A2). After a 24 h interval, during the testing session (T1B), the rats were allowed to explore one familiar object (A2) from the first session and a novel object (B) that had an altered shape, different enough to be distinguished. The objects were made of glass, plastic, and wood and were chosen after determining, in preliminary experiments with other animals, that they were equally preferred. The shapes, colors, and textures were different among these objects. Exploration of the objects was defined as sniffing or touching with the nose towards the object at a distance of less than 1 cm; however, sitting on the object was not considered exploration. Object placement was counterbalanced within each group in order to avoid any bias due to a preference that the rats may have had for a given object or its position in the box. The box and the objects were cleaned with 10% ethanol solution between each trial to remove traces of odor. A discrimination index (DI) was calculated, this being the ability to discriminate the novel from the familiar object: (novel object (s)/novel object (s) + familiar object (s) × 100%). Thus, an index > 50% indicates novel object preference, < 50%—familiar object preference, and 50%—no preference.

### Barnes maze task

The Barnes maze apparatus (Stoelting, Dublin, Ireland) consisted of a circular grey metal platform (diameter 122 cm), elevated 100 cm above the floor, with 20 holes (10 cm diameter) located on the periphery of the platform. Herein, one hole was connected to an escape box of 35 cm × 12 cm × 12 cm, of the same material and color as the platform. The other holes were covered underneath with a flat box so that the rats could not discriminate the escape hole from other holes (all the holes looked identical) until situated adjacent to it. Numerous visual cues (in the form of large colorful geometric shapes) were placed on the walls of the testing room at 1–2 m distance from the edge of the maze. To evoke the potentiated escape response, the platform was brightly lit (two points of light 1.5 m above the maze; 500 W each) and a buzzer (placed above the center of the maze) provided a sound of 80 dB as an aversive stimulus.

The Barnes maze task was run in 3 phases: habituation, acquisition and reversal trial. During the habituation trials, the rats were placed individually in the escape box for 2 min to acclimate to the box, and then were given time to explore the maze until they entered the escape box or 5 min had elapsed; if the rats did not enter the escape after 5 min, they were gently guided into the box. After spending an additional 15 s there, the rats were returned to their home cage. Habituation was performed with the lights on, but without the buzzer sound.

The same rats 24 h after the maze habituation, were subjected to the acquisition learning. Acquisition involved one training session per day for 3 consecutive days. Each training session consisted of two 180 s trials, with a 4-h inter-trial interval during which the animals were returned to their home cage. The location of the platform and the escape box remained constant over all the acquisition trials. Each trial began by placing the animal at the center of the platform, the buzzer was excited, and the rats were allowed to freely explore the apparatus. The trial was completed after 180 s or when the animal entered the escape box. Immediately after entering the escape box, the buzzer was switched off and the hole was covered for 15 s before the rat was returned to their home cage. If the animal did not enter the goal box within 180 s, it was gently guided there by the experimenter and could explore it for 15 s. On the fourth day, the animals were tested in two reversal trials. The reversal trial was identical to the acquisition trial, except that the position of the escape hole was rotated 180°. The rat was, therefore, unable to escape the maze using the acquired spatial cues, but had to relearn the new location of the hole. The data obtained from the acquisition trials and reversal trials was pooled together and used for calculations of primary latency and primary errors.

### Contextual and cued fear conditioning

During acquisition (training) the rats were placed into the fear conditioning chamber (55 × 60 × 57 cm; Ugo Basile, Italy) and were allowed to explore the chamber for 120 s during an acclimation period. Then, the rats were exposed to a sound signal (conditioned stimulus—CS) at a level of approximately 80 dB for 30 s. 0.5 mA foot shock (unconditioned stimulus—US) was given to the animals during the last 2 s of the sound. After a 120 s break, another analogous trial was performed. After this time, the rats remained for 60 s in the cage to associate and consolidate information. The chamber was cleaned with isopropyl alcohol in the periods in which the rats remained outside in order to retain the same fragrance. Contextual fear memory was assessed 24 h later by placing the rats back into the same chamber, but in the absence of tone and shock. After 120 s of adaptation, the animals remained there for 240 s during which the total time of freezing responses was assessed. The rats were not exposed to CS or US this day. As on the previous day, the cage was cleaned with isopropyl alcohol between trials.

Cued fear conditioning was measured 24 h after the contextual test. To measure cued fear learning, the rats were placed into a novel chamber (different floor and walls, the cage cleaned with isopropyl alcohol with the addition of vanilla extract) with the presentation of the tone but no foot shock. The rats were allowed 120 s to acclimatize followed by an exposition to a familiar sound stimulus (80 dB) for 3 min and the total time of freezing responses was evaluated. Afterwards, the sound was turned off and the animal remained in the cage for another 60 s.

### Statistical analysis

The obtained results were analyzed using GraphPad Prism version 8.00 for Windows (GraphPad Software, San Diego, California, USA). The data are reported as mean ± standard error of the mean (SEM) or median. Statistical significance of the data was assessed by a Chi-square test, Friedman test or repeated measures ANOVA (Statistica 12, StatSoft). *Post-hoc* comparisons were carried out with the Fisher’s test (ANOVA) test. Specific paired comparison was performed with Student’s *t*-test when necessary. A p-value of less than 0.05 was considered significant.

## Supplementary Information

Supplementary Figures.

Supplementary Tables.
