# Supplementary material for: Unexpected content of kynurenine in mother’s milk and infant formulas
Source: Sci Rep. 2022 Apr 19;12:6464. doi: 10.1038/s41598-022-10075-5 (PMC9018775; doi:10.1038/s41598-022-10075-5)
Supplement: Supplementary file 1 — Supplementary Figures. [file 41598_2022_10075_MOESM1_ESM.docx]

**A B**

**
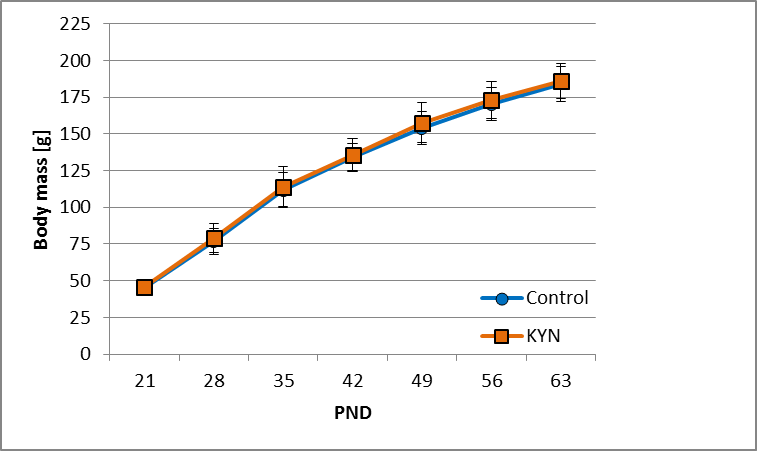

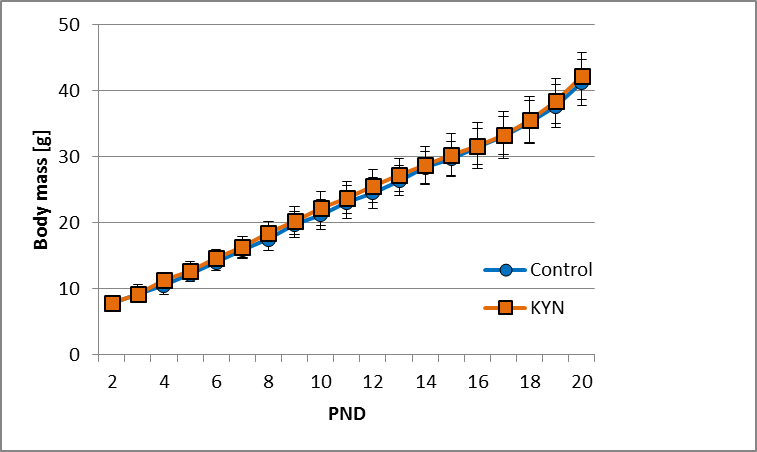

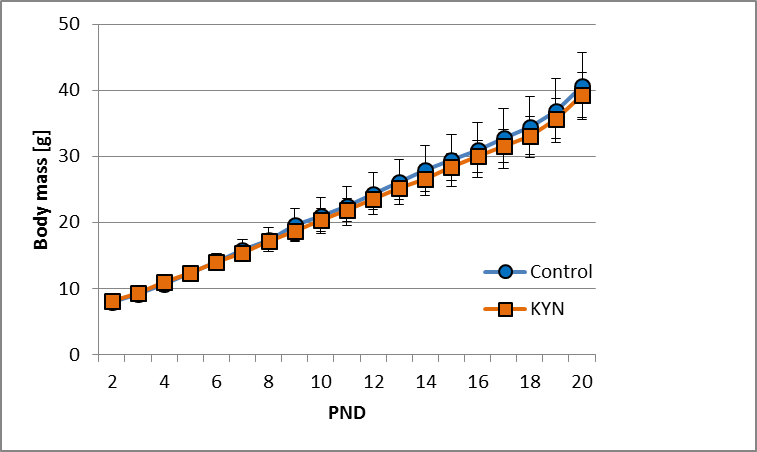

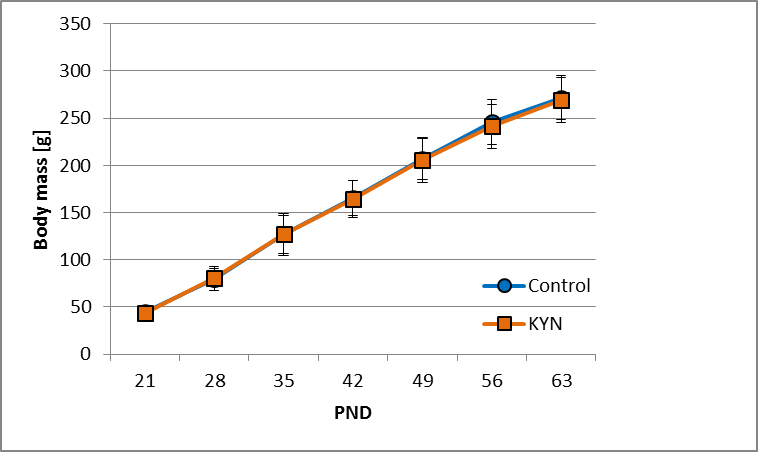
C D**

**Figure S1.** Effect of KYN administration during maternal feeding on body mass gain of male (A,B) and female (C,D) rats. Panel on the left (A,C) – from PND 2 to PND 20; panel on the right (B,D) – from PND 21 to PND 63. Data are presented as a mean ± SEM, number of subject = 10-12 per group, *P < 0.05 vs respective Control. KYN – kynurenine, PND- postnatal day.


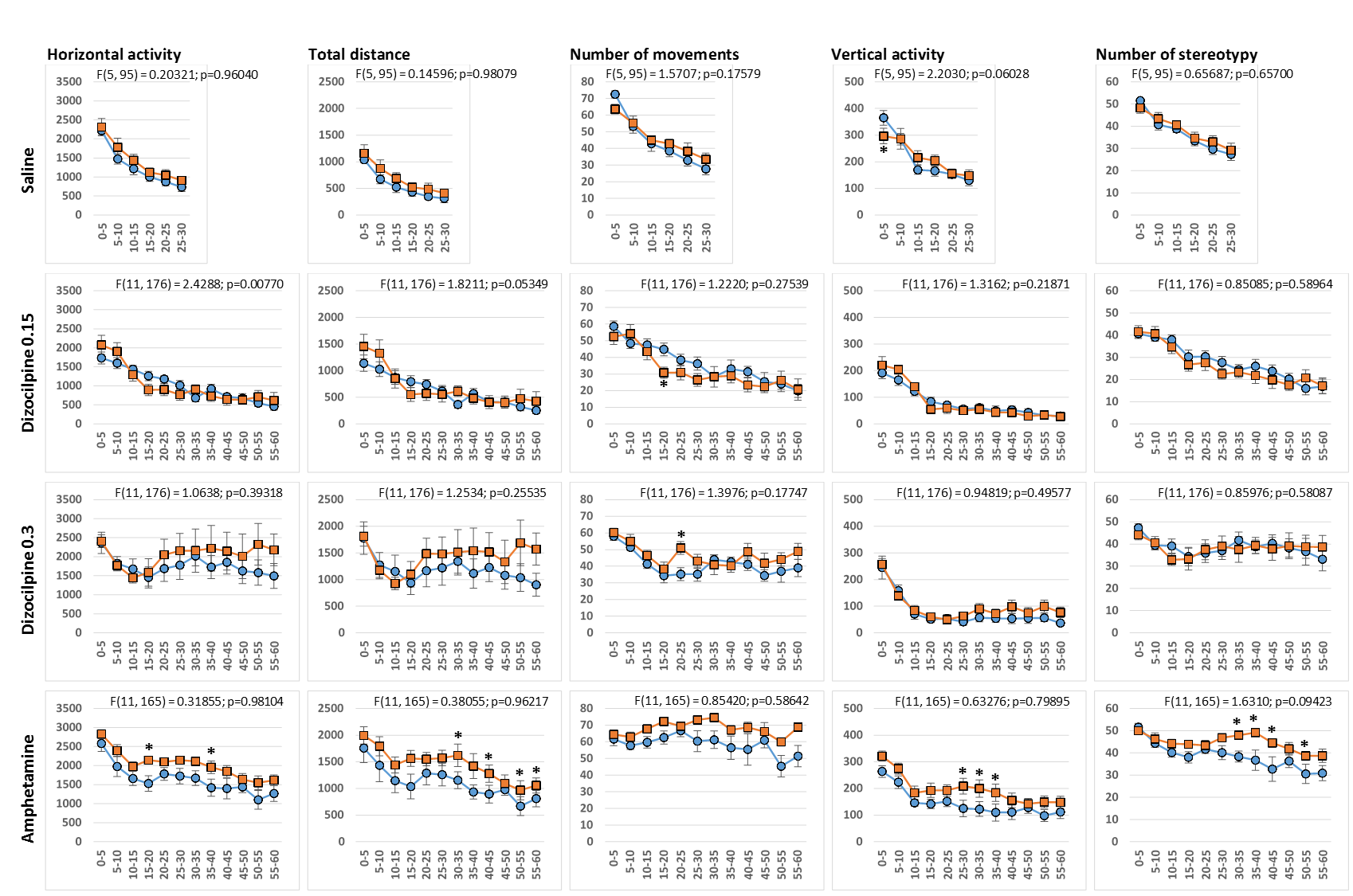


**Figure S2.** Effect of KYN administration during maternal feeding on spontaneous and stimulated locomotor activity of adult male rats. Data are presented as a mean ± SEM, number of subject = 10-12 per group, circles – Control, squares – KYN-treated rats. ANOVA with *post hoc* Fisher’s test, *P < 0.05 vs respective Control.


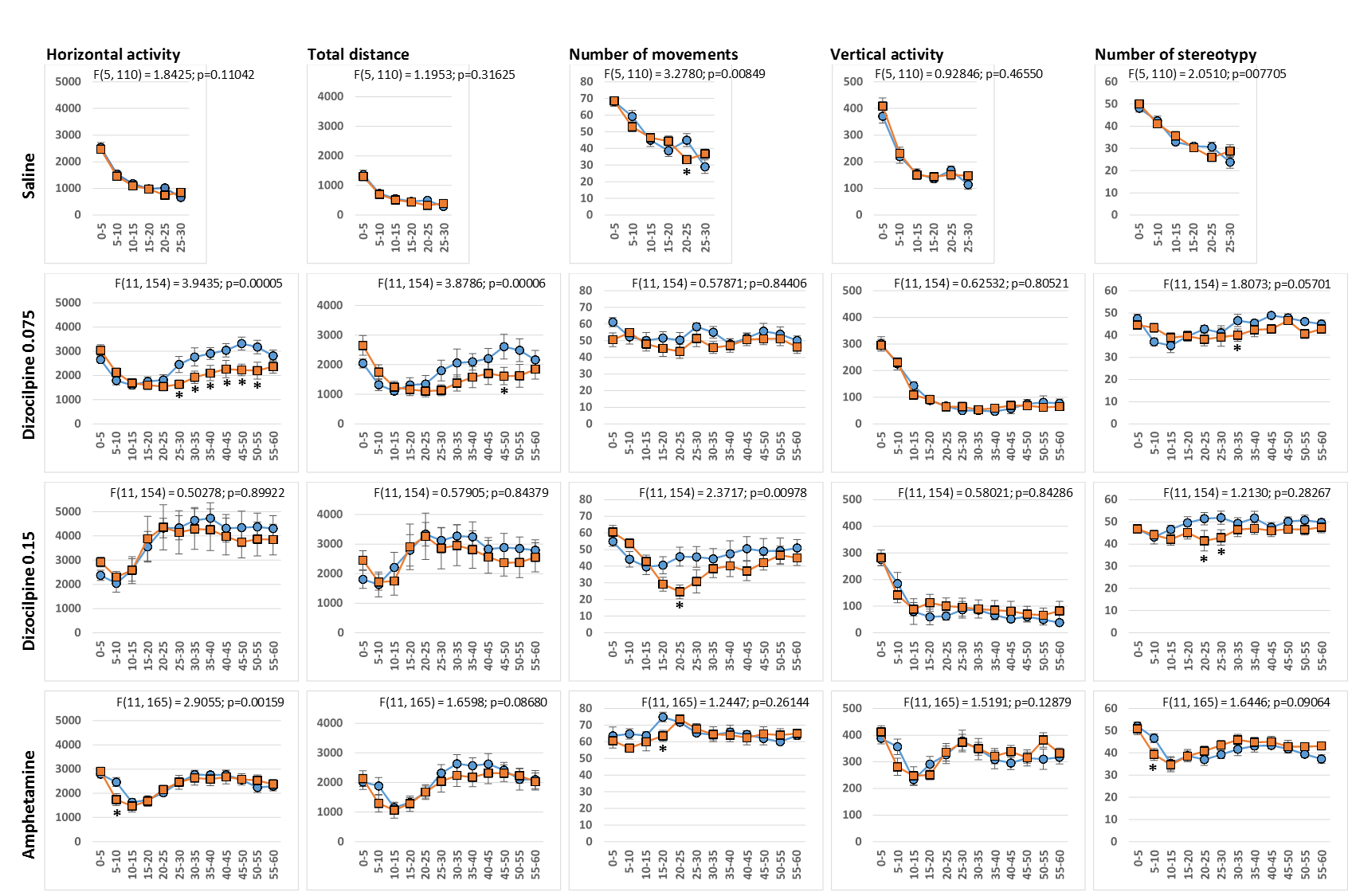


**Figure S3.** Effect of KYN administration during maternal feeding on spontaneous and stimulated locomotor activity of adult female rats. Data are presented as a mean ± SEM, number of subject = 10-12 per group, circles – Control, squares – KYN-treated rats. ANOVA with *post hoc* Fisher’s test, *P < 0.05 vs respective Control.

**Figure S4.** Effect of KYN administration during maternal feeding on anxiety-like behavior of adult rats estimated in the elevated plus-maze test. The number of entries into the open arms (A,B), the time spent by rats in the open arms (C,D), the number of total entries into the both open and closed arms as a read out of locomotor activity (E,F). Data are presented as a mean ± SEM, number of subject = 10-11 per group, *P < 0.05 vs respective Control, *t*-Student test. KYN – kynurenine.

**Figure S5.** Effect of KYN administration during maternal feeding on depressive-like behavior of adult rats estimated in the Porsolt swim test (A,B). Data are presented as a mean ± SEM, number of subject = 10-11 per group, *P < 0.05 vs respective Control, *t*-Student test. KYN – kynurenine.

**Figure S6.** Effect of KYN administration during maternal feeding on novel object recognition memory of adult rats. Memory performance (expressed as a discrimination index) (A,B), total exploration time (C,D). Data are presented as a mean ± SEM, number of subject = 9-10 per group, *P < 0.05 vs respective Control, *t*-Student test. KYN – kynurenine.
