# Supplementary material for: Unexpected content of kynurenine in mother’s milk and infant formulas
Source: Sci Rep. 2022 Apr 19;12:6464. doi: 10.1038/s41598-022-10075-5 (PMC9018775; doi:10.1038/s41598-022-10075-5)
Supplement: Supplementary file 2 — Supplementary Tables. [file 41598_2022_10075_MOESM2_ESM.docx]

**Table S1. Content of tryptophan and kynurenine in human milk.**

| Compound | Day | N | Median | Min | Max | 25% | 75% | Friedman ANOVA |
| --- | --- | --- | --- | --- | --- | --- | --- | --- |
| Tryptophan  [μg/mL] | 4 | 27 | 1.343 | 0.223 | 10.330 | 0.808 | 2.902 | Chi^2^=12.43  p=0.006 |
|  | 7 | 23 | 0.978 | 0.520 | 10.900 | 0.812 | 1.327 |  |
|  | 14 | 23 | 0.763 | 0.290 | 4.805 | 0.487 | 0.899 |  |
|  | 28 | 15 | 0.624 | 0.421 | 5.526 | 0.517 | 0.997 |  |
| Kynurenine [μg/mL] | 4 | 23 | 0.031 | 0.013 | 0.083 | 0.018 | 0.036 | Chi^2^=16.2  p=0.001 |
|  | 7 | 22 | 0.037 | 0.018 | 0.176 | 0.026 | 0.051 |  |
|  | 14 | 22 | 0.053 | 0.028 | 0.223 | 0.046 | 0.085 |  |
|  | 28 | 15 | 0.081 | 0.042 | 0.308 | 0.065 | 0.114 |  |

N – number of samples.

**Table S2. Content of kynurenine in plasma and liver tissue of 22-day old rats.**

| Gender | Group | Blood plasma [µg/mL] | Liver [µg/g wet tissue] |
| --- | --- | --- | --- |
| Male | Control | 0.24 ± 0.02 | 0.75 ± 0.08 |
|  | KYN | 0.23 ± 0.02 | 0.88 ± 0.10 |
| Female | Control | 0.22 ± 0.01 | 0.77 ± 0.13 |
|  | KYN | 0.19 ± 0.02 | 0.88 ± 0.11 |

Results are presented as a mean ± SEM; number of subject in each group = 10, *P < 0.05 vs respective Control, *t*-Student test. KYN – kynurenine.
